# Quorum Sensing *Pseudomonas* Quinolone Signal Forms Chiral Supramolecular Assemblies With the Host Defense Peptide LL-37

**DOI:** 10.3389/fmolb.2021.742023

**Published:** 2021-10-11

**Authors:** Ferenc Zsila, Maria Ricci, Imola Csilla Szigyártó, Priyanka Singh, Tamás Beke-Somfai

**Affiliations:** Institute of Materials and Environmental Chemistry, Research Centre for Natural Sciences, Hungarian Academy of Sciences (MTA), Budapest, Hungary

**Keywords:** circular dichroism, co-assemblies, host defense peptides, J-aggregates, LL-37, quorum sensing, supramolecular chirality

## Abstract

Host defense antimicrobial peptides (HDPs) constitute an integral component of the innate immune system having nonspecific activity against a broad spectrum of microorganisms. They also have diverse biological functions in wound healing, angiogenesis, and immunomodulation, where it has also been demonstrated that they have a high affinity to interact with human lipid signaling molecules. Within bacterial biofilms, quorum sensing (QS), the vital bacterial cell-to-cell communication system, is maintained by similar diffusible small molecules which control phenotypic traits, virulence factors, biofilm formation, and dispersion. Efficient eradication of bacterial biofilms is of particular importance as these colonies greatly help individual cells to tolerate antibiotics and develop antimicrobial resistance. Regarding the antibacterial function, for several HDPs, including the human cathelicidin LL-37, affinity to eradicate biofilms can exceed their activity to kill individual bacteria. However, related underlying molecular mechanisms have not been explored yet. Here, we employed circular dichroism (CD) and UV/VIS spectroscopic analysis, which revealed that LL-37 exhibits QS signal affinity. This archetypal representative of HDPs interacts with the *Pseudomonas* quinolone signal (PQS) molecules, producing co-assemblies with peculiar optical activity. The binding of PQS onto the asymmetric peptide chains results in chiral supramolecular architectures consisting of helically disposed, J-aggregated molecules. Besides the well-known bacterial membrane disruption activity, our data propose a novel action mechanism of LL-37. As a specific case of the so-called quorum quenching, QS signal molecules captured by the peptide are sequestered inside co-assemblies, which may interfere with the microbial QS network helping to prevent and eradicate bacterial infections.

## Introduction

The emergence of antimicrobial resistance (AMR) observed for multidrug-resistant bacteria forces a continuous search for suitable therapeutic agents against them. In this respect, bacteria display complex social behaviors and form communities of cells, called biofilms (Flemming et al., 2016), where they coordinate their activities through chemical communication. These biofilm matrices serve to protect the individual cells from the hostile environment and hence are key elements in AMR. As such a cooperative behavior is often dependent on the cell population density, the above-mentioned communication is usually referred to as quorum sensing (QS) (Miller and Bassler 2001). QS involves the microbial production and release of small organic molecules and oligopeptides, which are then recognized and used by others to monitor their population density and regulate gene expression accordingly (Wu and Luo 2021). Multiple interrelated mechanisms of QS systems allow the concerted actions of bacterial cells for the well-being of the entire population. Albeit QS was first described as *N*-acyl homoserine lactone-dependent cell-to-cell communication in Gram-negative bacteria (Coquant et al., 2020), QS signaling compounds exhibit significant chemical diversity. In view of developing novel antimicrobial therapies, the following features mediated by QS must be noted that make the pathogen robust: host colonization, adhesion, antibiotic and toxin production, acid tolerance, biofilm formation, motility, sporulation, and the secretion of virulence factors (Saxena et al., 2019; Azimi et al., 2020). All these demonstrate that biofilm eradication is of utmost importance in fighting AMR; nevertheless, despite many advances in treatment options, biofilm-associated infections are still a challenge to treat, requiring either large doses of antibiotics or surgical removal of the infected medical devices.

A natural set of compounds bearing antibiofilm potential are host defense peptides (HDPs), also commonly termed antimicrobial peptides (AMPs) (Di Somma et al., 2020). HDPs are typical innate immune effector molecules expressed by a variety of different cell types and are found throughout the body (Alba et al., 2012; Mookherjee et al., 2020). They are small, typically 10–50 amino acids long, showing cationic nature due to the relative abundance of basic residues. Mostly by perturbing microbial membranes, these agents are well known for their ability of direct killing of many infectious agents. Their actions also involve a variety of inflammatory and immunomodulatory responses (Alba, Lopez-Abarrategui and Otero-Gonzalez 2012; Hancock et al., 2016). Moreover, besides their monomeric form, an increasing amount of data indicate that their supramolecular self- or co-assembled scaffolds can also have functional relevance (Lee et al., 2019; Engelberg and Landau 2020; Ricci et al., 2020; Quemé-Peña et al., 2021). It has also been shown that various small organic molecules as well as lipid clusters trigger the disorder-to-order conformational transition of intrinsically unfolded AMPs, facilitating their oligomerization and complex formation, which can also affect the peptide function *in vivo* (Ricci et al., 2020; Quemé-Peña et al., 2021). Importantly, similar interactions were observed between a wide range of membrane active AMPs and lysophosphatidic acid (LPA) (Juhász et al., 2018), a human lipid signal with diverse functions including stimulation of cell migration and proliferation (Benesch et al., 2018). It should be noted that numerous HDPs show much higher affinity for degrading biofilms than killing individual bacteria, which is potentially achieved by interfering with QS (Grassi et al., 2017; Koo et al., 2017). Although the interaction network between HDPs and biofilms and also related virulence factors is clearly a complex phenomenon, occurring on several levels (Overhage et al., 2008; Strempel et al., 2013), it is particularly intriguing and highly relevant to address whether AMPs could act in inhibition of QS and related processes by direct interactions with bacterial signaling molecules. As the molecular basis for the above antibiofilm action of HDPs is so far not revealed, here, we employed biophysical studies to explore interactions between HDPs and QS molecules.

**SCHEME 1:**
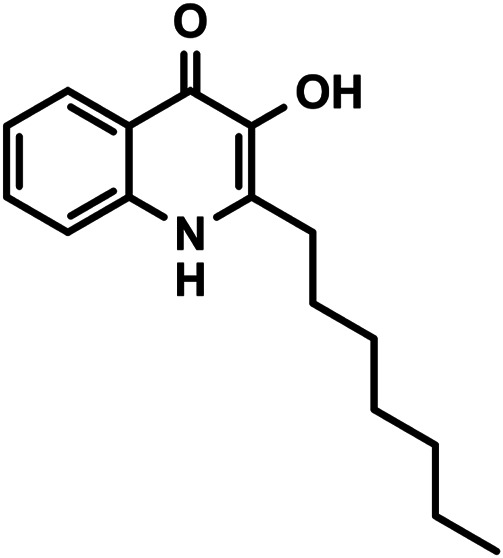
Chemical structure of PQS (2-heptyl-3-hydroxy-4(1*H*)-quinolone).

An important representative of HDPs is LL-37, the sole human cathelicidin family member, which is named for consisting of 37 amino acids, with two N-terminal leucines (Durr et al., 2006; Zeth and Sancho-Vaello 2017). Its sequence is enriched in lysine and arginine residues, resulting in a +6 net charge under physiological conditions (LLGDFFRKSKEKIGKEFKRIVQRIKDFLRNLVPRTES). LL-37 can be detected in a number of tissues and body fluids where it is produced by epithelial cells, monocytes, polymorphonuclear leukocytes, NK, and B-cells (Durr et al. 2006; Vandamme et al., 2012). Beyond direct antimicrobial activities, the biological functions of LL-37 extend into broad fields such as chemoattraction (Wong et al., 2013), promotion of cytokine production (van Harten et al., 2018), wound healing (Mangoni et al., 2016), carcinogenesis (Chen et al., 2018), regulation of gut microbiota (Zhang et al., 2019) and also proved antibiofilm activity against the ESKAPE pathogens (*Enterococcus faecium, Staphylococcus aureus, Klebsiella pneumoniae, Acinetobacter baumannii, Pseudomonas aeruginosa*, and *Enterobacter* species), although with an unknown mechanism (Mishra and Wang 2017). The Gram-negative *P. aeruginosa* is a well-known opportunistic, often a multidrug-resistant human pathogen, that presents a major threat for immunocompromised patients as its affinity for extensive colonization quickly results in biofilm formation, giving rise to persistent infections (Jurado-Martin et al., 2021). One of its three interconnected QS networks uses the secondary metabolite *Pseudomonas* quinolone signal (PQS) (Scheme 1) (Sams et al., 2016; Lin et al., 2018). After being secreted into the host environment, PQS and related compounds may interact not only with their specific bacterial receptors but also with the component of the innate immune system. As an initial investigation on potential molecular level interactions taking place between QS components and HDPs, we have selected PQS and LL-37 and screened the circular dichroism (CD) and UV/VIS absorption spectra of the former admixed to the latter with an aim to identify spectral traces of small molecule–peptide interactions. The observed spectroscopic signatures were evaluated in detail to deduce structural information on this unique case of host-microbial molecular interplay. The results indicate that LL-37 has the capacity to form supramolecular associates with PQS, which proposes that the molecular basis of antibiofilm peptide activity, at least in part, could be related to QS perturbation, where entrapment of the key molecules results in the disruption of bacterial biofilms and thus contributes to antibacterial activities.

## Materials and Methods

### Materials

PQS (2-heptyl-3-hydroxy-4(1*H*)-quinolone) was purchased from Sigma-Aldrich (product no. 94398, purity 99.8%). C-terminal amidated LL-37 (LLGDFFRKSKEKIGKEFKRIVQRIKDFLRNLVPRTES-NH_2_, cat. no. 308114) was synthesized by NovoPro Bioscience Inc. (Shanghai, China). Peptide purity corresponded to 96.20%. The molecular weight of the peptide (4492.25) was determined by mass spectrometry. The net peptide content of the supplied material was 72.12%. All other chemicals were of analytical reagent grade.

### Preparation of Peptide and the *Pseudomonas* Quinolone Signal Solutions

LL-37 solutions were prepared in pH 7.4 phosphate-buffered saline (PBS) or in 10 mM Tris-HCl buffer (pH 7.4). PBS was prepared by dissolving 8 g of NaCl (137 mM), 0.2 g of KCl (2.7 mM), 1.8 g of Na_2_HPO_4_·2H_2_O (10 mM), and 0.312 g of NaH_2_PO_4_·2H_2_O (2 mM) in 1 L of Milli-Q water. PQS was dissolved in spectroscopic grade ethanol.

### Circular Dichroism and UV/VIS Absorption Spectroscopy

Circular dichroism (CD) and UV/VIS absorption spectra were recorded on a JASCO J-715 spectropolarimeter at 25 ± 0.2°C in a 0.2 cm path-length rectangular quartz cuvette (Hellma, United States). Temperature control was provided using a Peltier thermostat. CD data as ellipticities in mdeg were collected in a continuous scanning mode between 200 and 460 nm at a rate of 100 nm/min, with a step size of 0.1 nm, a response time of 2 s, three accumulations, and a 1 nm bandwidth. CD curves of peptide and PQS-peptide samples were corrected by the spectral contribution of the blank PBS and the free LL-37, respectively. For CD titration measurements, 400 μL of LL-37 solution (33–34 μM) was placed in the cuvette. After finishing the CD scans, μL aliquots of the PQS stock solution were pipetted consecutively into the cuvette and the CD data were collected after each aliquot. For the evaluation of the structural effect of ethanol added with PQS during the titration procedure, the far-UV CD curves of LL-37 were measured at increasing ethanol concentrations. UV/VIS absorption spectra of PQS in the absence and presence of LL-37 were recorded by conversion of the high-tension (HT) values of the photomultiplier tube of the CD equipment into absorbance units. All CD and absorption spectroscopic measurements were implemented once.

### UV/VIS Spectroscopic Measurements for Determination of the Critical Aggregation Concentration of the *Pseudomonas* Quinolone Signal

UV/VIS spectra were recorded using a Hewlett-Packard 8453 diode array spectrophotometer at 25°C in PBS between 190 and 600 nm in 1 nm increments using a quartz cuvette of a 1 cm path length. The titration experiment was performed by a consecutive increase of PQS concentration up to 15 μM. All spectra were baseline-corrected with the spectrum of PBS. UV/VIS spectroscopic measurements were implemented twice.

### Dynamic Light Scattering Experiments

The average size, size distribution, and time-dependent autocorrelation functions of the formed aggregates were evaluated using a W130i apparatus (Avid Nano Ltd., High Wycombe, United Kingdom). Samples containing a constant peptide concentration (34 μM) and various amounts of added PQS were measured in PBS in a final volume of 200 μL in low-volume disposable cuvettes (UVette, Eppendorf Austria GmbH). The analyses of the measurements were performed using the i-Size 3.0 software supplied with the device.

### Computational Details

All computations were carried out using the Gaussian 09 software package (Frisch et al., 2004). To optimize CPU requirements, the PQS molecule was truncated, keeping only its aromatic hydroxyquinolone moiety. The structure was optimized at the ωB97X-D/6-311++G (2d, 2p) level of theory. To aid identification of which electronic transition dipole moment (etdm) belongs to which band in the experimentally observed spectra as well as to obtain the orientation of these moments within the molecule, excited energies and UV/VIS spectra of the optimized geometry were calculated using time-dependent density functional theory (TD-DFT) calculations at the same level of theory as indicated above. Accordingly, the three etdms with the highest oscillator strength were found at 341.04, 239.68, and 223.05 nm.

## Results and Discussion

### UV/VIS Absorption Spectroscopic Features of the Aqueous Aggregation of the Peptide-Free *Pseudomonas* Quinolone Signal

**FIGURE 1:**
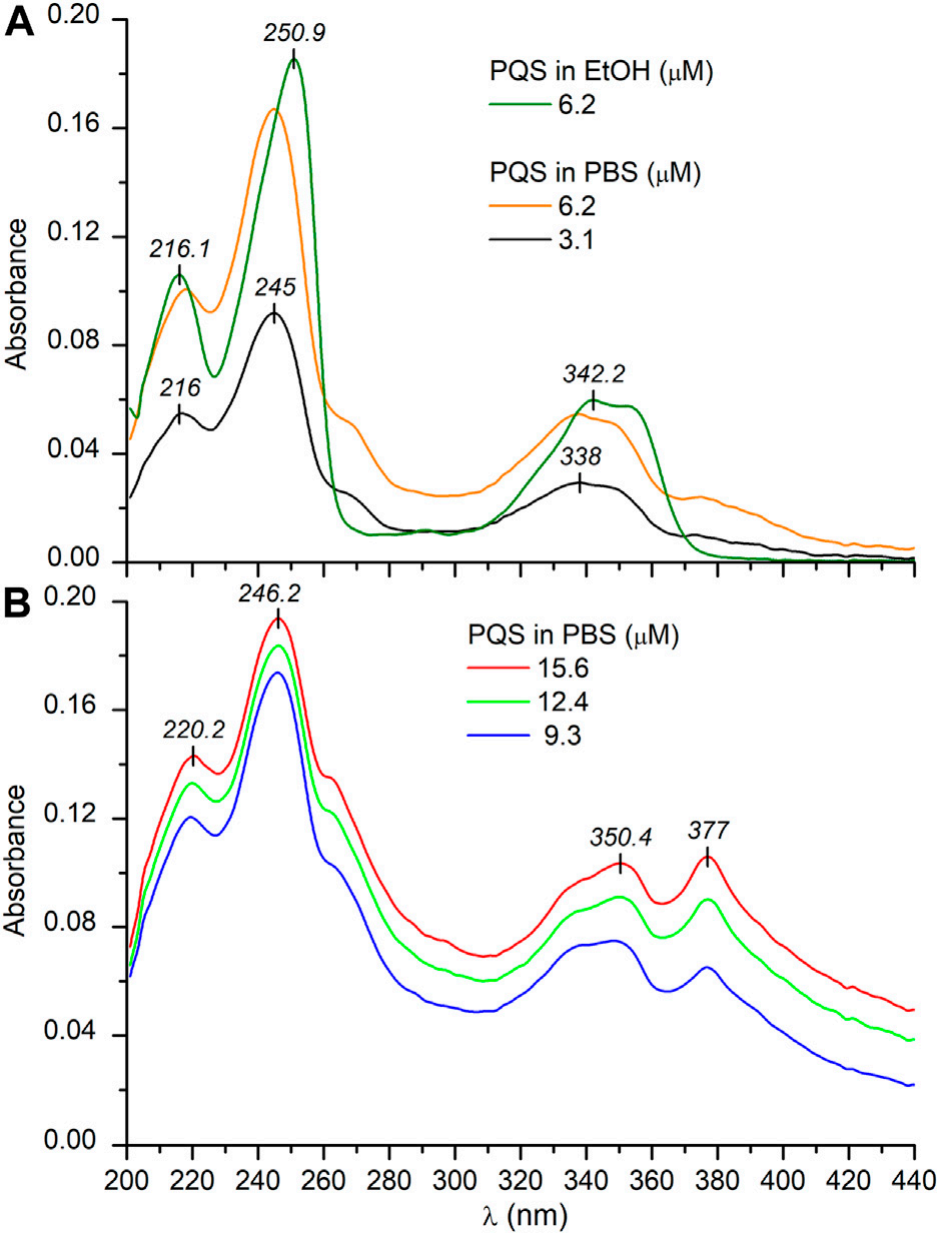
UV/VIS absorption spectra of PQS recorded in PBS at low **(A)** and at higher **(B)** concentrations. The absorption curve obtained in EtOH is also shown **(A)**. The optical path length is 1 cm.

**FIGURE 2:**
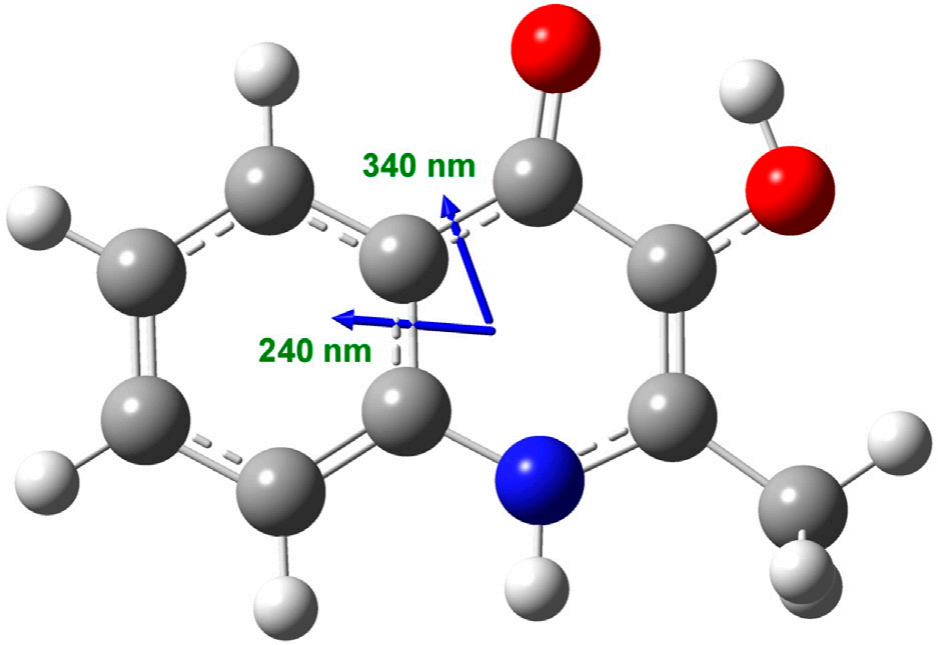
Calculated direction of the π–π^*^ electronic transition dipole moments in the aromatic segment of the PQS molecule coupled to the ∼240 and ∼340 nm excitations as obtained at the ωB97X-D/6-311++G (2d, 2p) level of theory.

The planar, hydroxyquinolone chromophore of PQS is responsible for the multicomponent absorption band system measured in PBS above 200 nm (Figure 1A). The most intense peak is centered at 245 nm overlapping with two adjacent, weaker bands at both the shorter and longer-wavelength sides. A broad, less intense band is located between 300 and 370 nm decorated with two, partially resolved maxima. In relation to the buffer solution, a similar absorption spectroscopic profile was obtained in ethanol with some differences to be noted. In the organic solvent, a moderate red shift of the *λ*_max_ values can be observed. Additionally, no absorption band is displayed either above 375 nm or between 260 and 280 nm (Figure 1A). Taking into consideration the structure of PQS as well as its molar absorption intensities (*ε*_max_), the UV absorption bands can be assigned to the *π*–*π* ^*^ transitions of the conjugated *π*-system of the planar hydroxyquinolone moiety. To determine the respective electronic transition dipole moments (etdms) associated with the main absorption peaks, TD-DFT quantum chemical calculations were performed. According to the results, the etdm of the higher oscillator strength 240 nm band lies along approximately the long axis of the ring system, whereas for the longer-wavelength transition, it is oriented at 70° relative to the direction of the 240 nm etdm (Figure 2).

**SCHEME 2:**
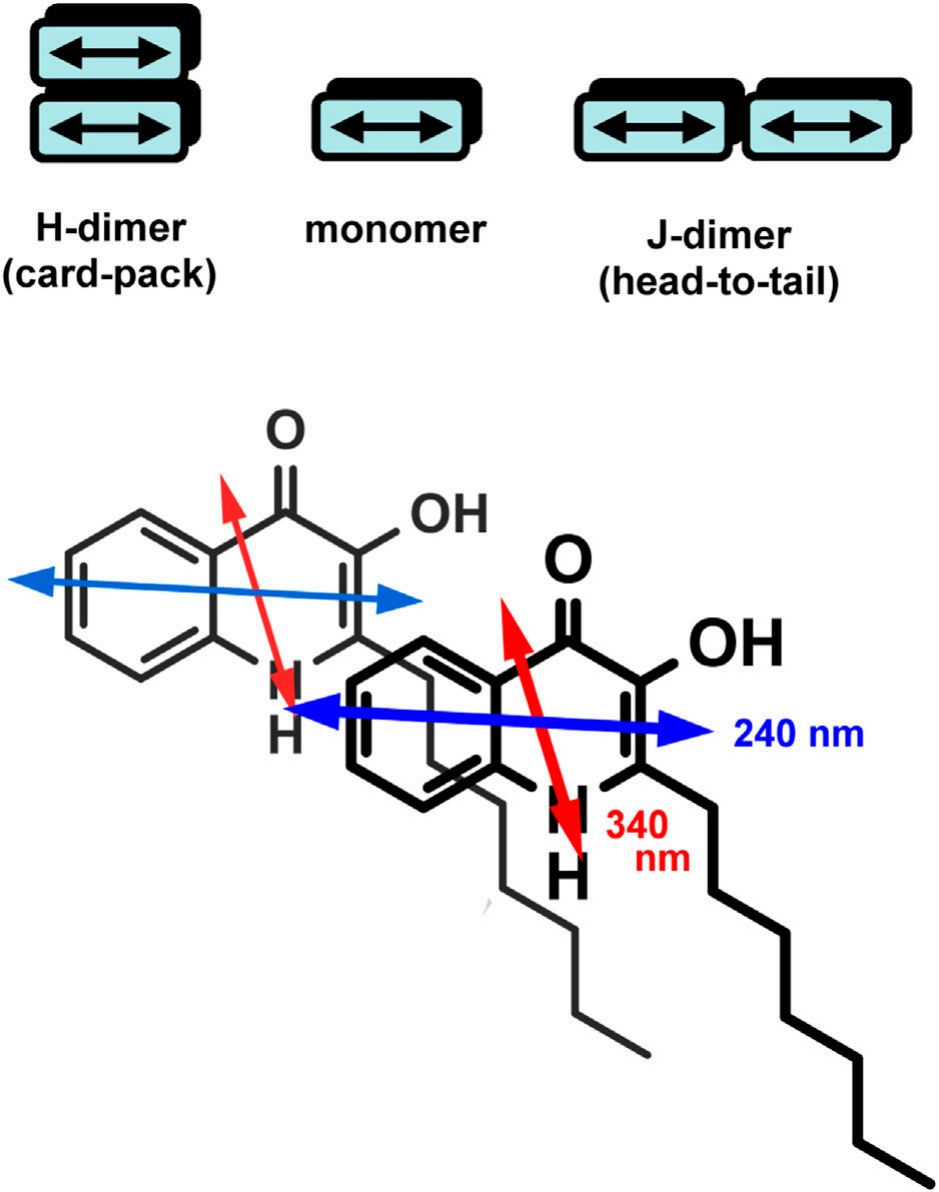
Top: illustration of aggregated dimers in H- and J-type arrangement. Per molecule, one electronic transition dipole moment is shown (double-headed arrow). Bottom: a hypothetical J-dimer formed by two PQS molecules. Note the head-to-tail relative orientation of the respective etdms.

Upon increasing the PQS concentration above 3 μM, the UV/VIS spectra exhibited some notable alterations. At the red edge of the spectrum, the zero baseline started to elevate, and it is obvious that the absorbance values do not follow the Beer–Lambert law (Figure 1B). The amplitude of the peak at 245 nm hardly increases with the concentration, whereas the unresolved band around 260 nm gained double intensity. Similarly, the longest-wavelength weak and broad band above 360 nm strongly increased and finally became equal in magnitude with the adjacent peak. In concert with these changes, increased opalescence of the sample solution could be noticed. In view of the observations, the anomalous absorption spectroscopic features can be attributed to the concentration-dependent, progressive aqueous aggregation of the hydrophobic PQS molecules. Associative interactions between planar organic compounds possessing limited water solubility such as acridine (Ikkda and Imae 1971) and cyanine dyes (Kim et al., 2006; Wurthner et al., 2011), carotenoids (Zsila et al., 2001), and porphyrin derivatives (Kubat et al., 2003) occur frequently in aqueous solutions. Depending on the particular chemical constitution of the molecules, the resulting dimers, oligomers, and higher-order aggregates may be stabilized by *π*–*π* stacking and attractive coulombic and/or van deer Waals forces. Packing of the aromatic rings within PQS associates results in the delocalization of the *π*–*π* ^*^ excited states over a wide range of the adjacent chromophores. Thus, electronic absorption spectra of such optically excited aggregate systems display characteristic signs due to the intermolecular electronic interaction between the excited states termed as exciton coupling (Kasha 1963; Kasha et al., 1965; Boiadjiev and Lightner 2005). In relation to the absorption features of the monomeric state, spectral changes caused by exciton coupling include hypo- or hyperchromism of existing bands, red or blue shift of the *λ*_max_ values, band broadening/narrowing, and/or the development of one or more additional peaks. Depending on the relative arrangement of the molecules established inside the assemblies, the resulting spectral signatures may indicate the existence of J- or H-type aggregation (Zsila et al., 2001; Kim et al., 2006; Wurthner et al., 2011; Pescitelli et al., 2014). Compared to the monomeric compound, J-aggregates exhibit a new, red-shifted, narrow, and intense band due to the parallely displaced, head-to-tail-type geometry of the chromophores enabling intermolecular excitonic delocalization between them (Scheme 2). In concert with this, the evolution of new, red-shifted peaks of PQS centered at 265 and 377 nm (Figure 1B) is indicative of the presence of J-aggregating motifs composed of hydroxyquinolone moieties. A hypothetical J-dimer of two PQS molecules is shown in Scheme 2 stabilized by CH–*π* interactions formed between the alkyl chain and the *π*-system of the hydroxyquinolone rings. Note that this kind of arrangement ensures exciton coupling between the head-to-tail oriented etdms, resulting in red-shifted J-bands in the absorption spectrum at 265 and 377 nm (Figure 1B). The steadily growing light scattering of the solution as well as the spectral overlap with the still significant monomeric absorption render these new bands in part unresolved. The weak bands in the UV/VIS spectrum at 264 and 377 nm recorded even at the lowest PQS concentration suggest that a minor fraction of the molecules are already in an aggregated state even in dilute solution (Figure 1A). The evaluation of the absorption curve measured in ethanol lends further credence to this assumption. The complete absence of these bands indicates pure monomeric state of PQS in that solvent.

Further on, UV/VIS spectroscopic measurements were performed to determine the critical aggregation concentration of PQS in PBS solution. At low concentrations (∼1–2 μM) only monomers are present in the buffer solution (Supplementary Figure S1). With increasing concentration, the J-band appears with a maximum at 376 nm which could be assigned to the aggregate formation. Considering the UV signal enhancement, titration data showed nearly linear dose dependence, with a breakpoint in the lower-concentration range (Supplementary Figure S1, inset). Similar to micellar systems, PQS molecules dissolved in PBS are monomeric below the critical aggregation concentration (cac), an important parameter that characterizes the self-assembling behavior of such systems. The cac value was estimated from the discontinuity in the absorbance intensities at 376 nm, resulting in a 2.94 ± 0.34 μM value in PBS (Supplementary Figure S1, inset).

### Conformational Properties of LL-37 in Buffer Solutions

**FIGURE 3:**
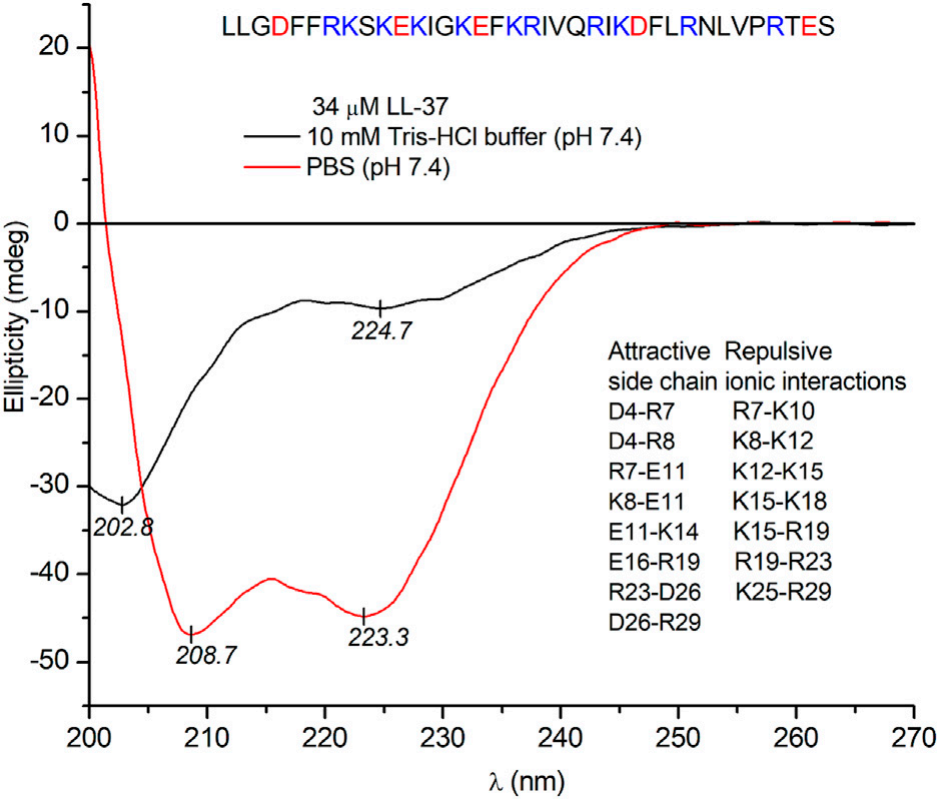
Far-UV CD spectra of 34 μM LL-37 recorded in PBS and in a salt-free Tris-HCl buffer solution (the optical path length is 0.2 cm). PBS contains 137 mM NaCl and 2.7 mM KCl. The sequence of the peptide and helix promoting attractive and destabilizing repulsive electrostatic side chain interactions are shown (see text).

Evaluation of the far-UV CD spectra is an excellent and rapid tool to obtain valuable information about conformational features of the peptide backbone, that is, the relative contribution of the most important secondary structural elements such as *α*-helix, *β*-sheet, and random coils (Toniolo et al., 2012). Therefore, the secondary solution structure of LL-37 was investigated by CD spectroscopy. In line with previous reports (Brannon et al., 2015; Johansson et al., 1998) and consistent with the two negative extrema at 209 and 223 nm, the far-UV CD spectrum of LL-37 obtained in PBS solution refers to the ordered, primarily *α*-helical conformation of the peptide chains (Figure 3). In contrast, the CD curve recorded in a salt-free Tris-HCl buffer (pH 7.4) lacks the helix-like, similar intensity double minima and shows a main negative peak of *π*–*π* ^*^ origin centered at 203 nm, whereas the *n*–*π* ^*^ transitions of the amide chromophores produce a weaker, broad band having a minimum around 225 nm (Figure 3). This CD pattern is fairly typical of a predominantly disordered state with a minimal helix content (Johansson et al., 1998; Zsila et al., 2019). Accordingly, the *α*-helical structure of LL-37 maintained in PBS is completely disrupted in the absence of inorganic anions. The balance of intrapeptide ionic attractions–repulsions between charged residues (arginine/lysine and glutamate/aspartate) might be a decisive factor in determination of the helix-coil equilibrium, particularly for sequences rich in acidic and basic amino acids. Salt bridges formed by acidic and basic residues located in positions *i* and *i* + 3 or *i* + 4, which are close on the *α*-helix, specifically stabilize the helical conformation (Johansson et al., 1998; Scholtz et al., 1993). *Vice versa*, side chains with the same charge located at these sites destabilize the helix due to the repulsive electrostatic interactions. The sequence of LL-37 contains nine potential helix-promoting oppositely charged ion pairs and seven combinations of basic residues located both in positions *i* and *i* + 3 or *i* + 4 (Figure 3). As inferred from CD spectroscopic data, the presence of various anions in a suitable concentration shifts the conformational equilibrium of LL-37 toward the ordered, helical form (Johansson et al., 1998; Zsila et al., 2019). The anion-induced folding may occur *via* coulombic shielding of the cationic side chains, thereby reducing the repulsion forces between them.

### Circular Dichroism and Absorption Spectroscopic Evaluation of LL-37-*Pseudomonas* Quinolone Signal Interactions

**FIGURE 4:**
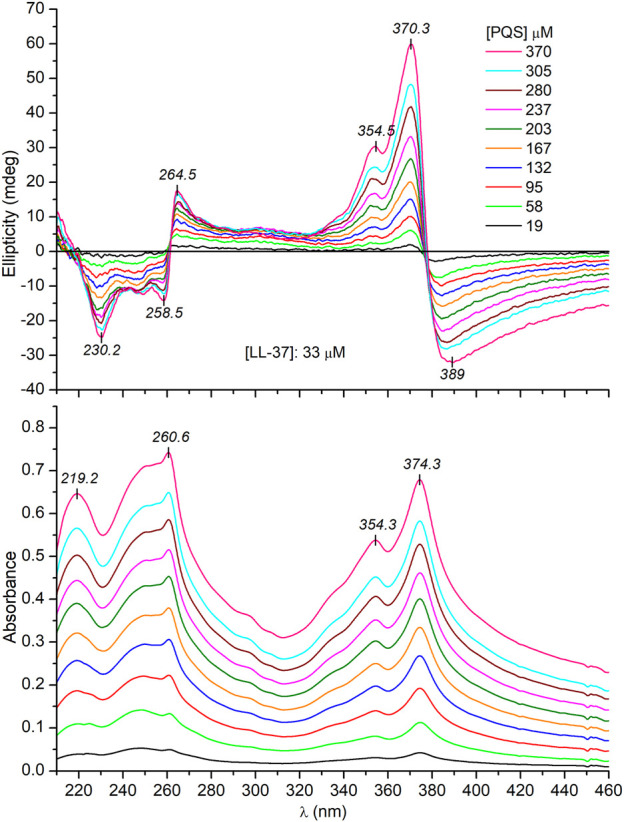
Representative CD and UV/VIS absorption spectra recorded upon a consecutive increase of PQS concentration in PBS solution of 33 μM LL-37 (the optical path length is 0.2 cm).

**SCHEME 3:**
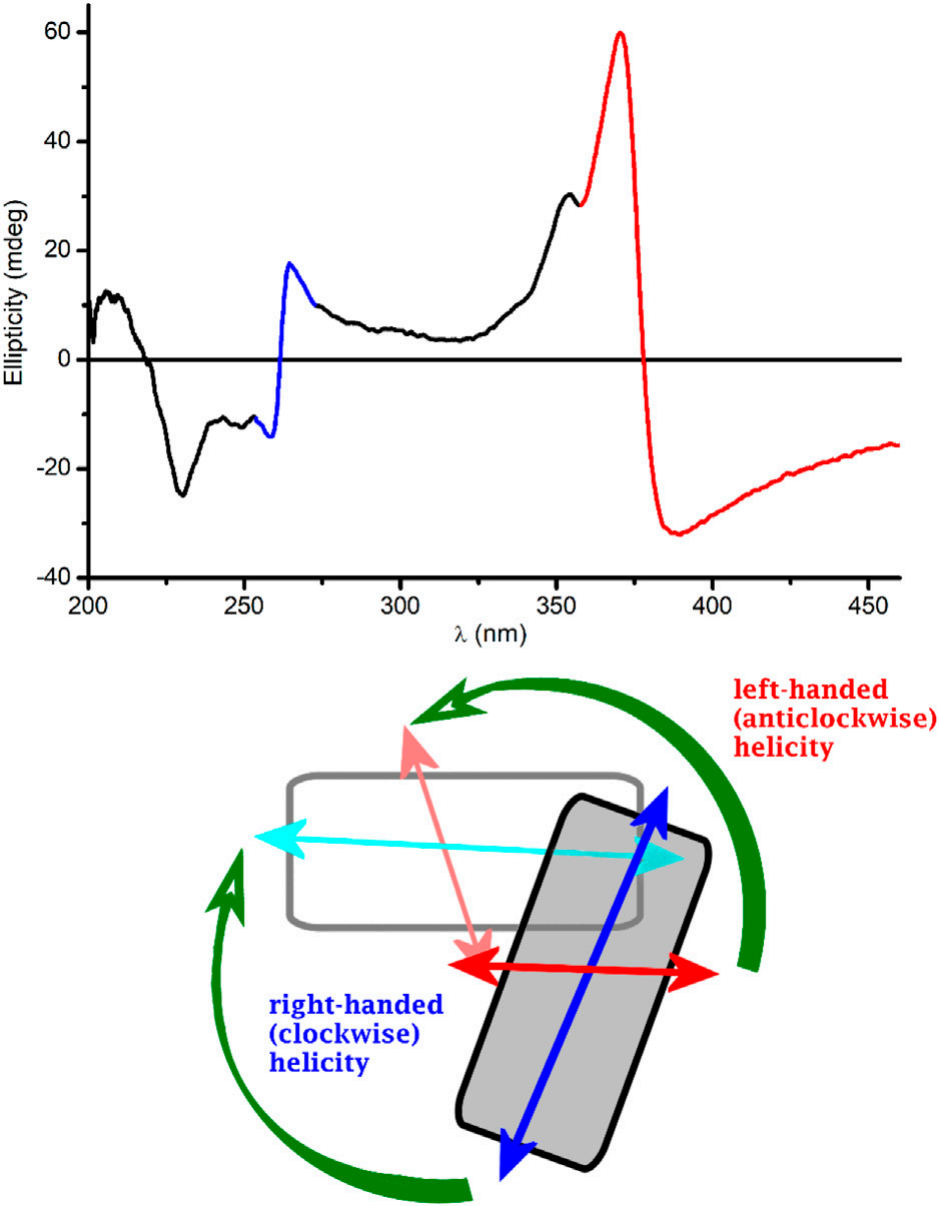
Possible twisted disposition of two J-dimers composed from pairs of PQS molecules. For clarity, the dimers are represented by rectangles. The gray-colored dimer is above the plane of the paper. Double-headed arrows denote the vectorial sum of the corresponding etdms (blue and light blue for 240 nm, red and light red for 340 nm). Chiral exciton coupling between dimer etdms results in a negative–positive (left-handed) and a positive–negative (right-handed) CD exciton couplet (red- and blue-colored segments of the experimental CD curve, respectively).

Since PQS is achiral, neither its monomeric form (in EtOH) nor its self-associated form (in buffer) exhibits CD signals (data not shown). Surprisingly, upon addition of PQS to LL-37 dissolved in PBS, well-resolved multiple CD bands were induced (Figure 4). The respective absorption bands to which these signals are allied bear the typical spectroscopic features of the aggregated state. Similar to that observed in a peptide-free buffer solution, two new peaks appeared around 260 and 375 nm and the long-wavelength tail of the absorption curve rose strongly with the increase of PQS concentration. The band developed at 375 nm splits into two intense Cotton effects (CEs) of opposite signs in the CD spectrum, changing from negative to positive close to the absorption maximum (Figure 4). At shorter wavelengths, a similar but oppositely signed and less intense CD couplet is shown associated with the UV maximum at 260 nm. Additional negative signals are displayed around 248 and 230 nm. The magnitude of the induced CEs increases with the PQS concentration of the sample solution but cannot be saturated even at 15-fold molar excess of the ligand molecules. According to the induced CD pattern observed above 240 nm, the peptide chains serve as asymmetric templates for PQS molecules to build up chiral supramolecular aggregates. It is reasonable to assume that in the initial phase, the association of J-aggregated PQS dimers/oligomers to LL-37 occurs in a chiral fashion dictated by the asymmetric centers of the side chains. In parallel with the advance of ligand loading, the size of the chiral-arranged associates progressively increases by the adsorption of more and more apolar signal molecules. It should be noted that the parallel alignment of adjacent PQS molecules (Scheme 2) lacks any chiral organization. Thus, it is proposed that the induced CD activity stems from the twist between J-aggregated building blocks deposited onto the chiral surface of LL-37. According to this mechanism, chiral exciton coupling between such helically disposed J-aggregated dimers or oligomers is responsible for the bisignate CEs observed experimentally (Kim et al., 2006). The helical sense of the peptide-templated self-assemblies can be deduced from the exciton chirality rule (Boiadjiev and Lightner 2005; Pescitelli et al., 2014). The in-phase and out-of-phase exciton coupling of the individual *π*–*π* ^*^ transition moments of hydroxyquinolone chromophores arranged helically relative to each other in the assembly generate two bands, a higher- and a lower-energy oppositely signed CD band. The sign order of these exciton CEs is governed by the relative orientation of the interacting transition moments: a clockwise screw sense between them leads to a longer-wavelength positive CD band and a shorter-wavelength negative CD band and *vice versa*. Taking this into consideration, the main bisignate CD feature allied to the J-band in the near-UV region suggests the prevalence of counterclockwise twist between the interacting transition dipole moments oriented along the long axis of the hydroxyquinolone ring. In view of the shorter-wavelength opposite CD couplet, the large deviation of its respective etdm from that of the 340 nm band must be considered (Figure 2). This implies that various intermolecular arrangements exist where these etdms are coupled just in an opposite sense in relation to the longer-wavelength dipole moments (Scheme 3). Consequently, the prevalence of these species can be accounted for the reversed, negative–positive and positive–negative exciton couplets measured for the J-absorption band at 375 and 261 nm, respectively (Figure 4).

Besides the exciton signals, an additional positive CE is displayed at 354 nm. It coincides with the UV band centered at the same wavelength which represents the light absorption of the monomeric PQS species (Figure 4). Accordingly, a monomeric fraction of PQS is also involved in the peptide binding and thus gives contribution to the induced CD activity presumably via the non-degenerate exciton coupling mechanism (Zsila et al., 2011).

**FIGURE 5:**
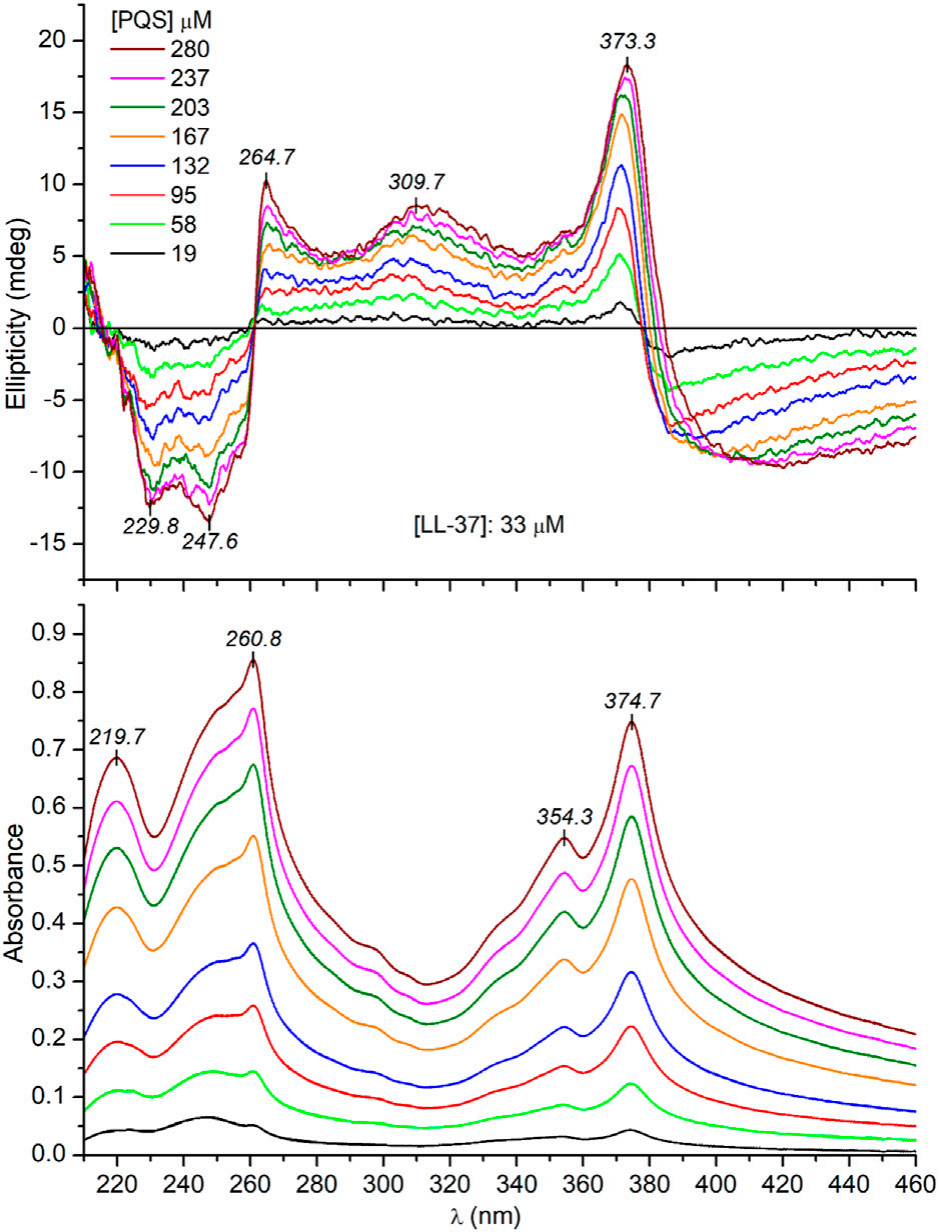
Representative CD and UV/VIS absorption spectra recorded upon a consecutive increase of PQS concentration in Tris-HCl buffer solution of 33 μM LL-37 (the optical path length is 0.2 cm).

**FIGURE 6:**
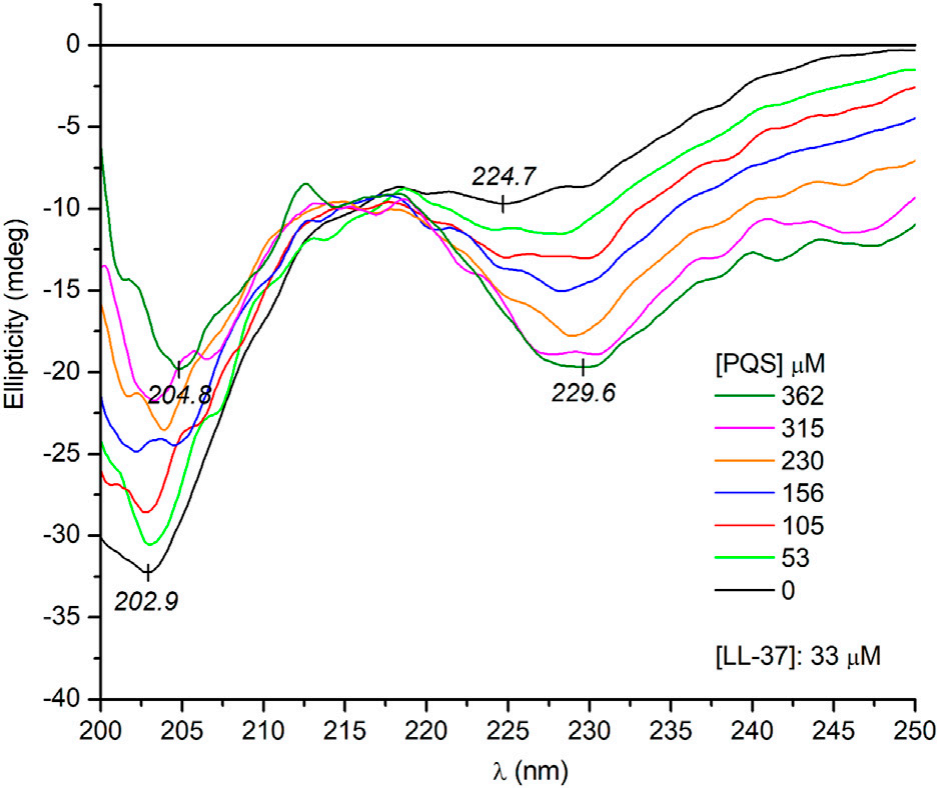
Far-UV CD spectra of LL-37 at increasing PQS concentrations recorded in a salt-free Tris-HCl buffer solution (the optical path length is 0.2 cm).

It is tempting to speculate that the supramolecular chirality of the J-aggregates is somehow determined and favored by the *α*-helical conformation of the peptide chains. In this case, the unordered form of LL-37 being prevalent in a salt-free Tris-HCl buffer (Figure 3) should induce no or completely different CD motifs. Interestingly, this is not the case since very similar induced exciton CEs were measured to those obtained in PBS solution (Figure 5). One possible reason for this result is that PQS binding promotes the disorder-to-helix conformational conversion of LL-37 as it has been observed previously with various pharmaceutical agents, food dyes, and porphyrin metabolites (Zsila et al., 2018; Zsila et al., 2019). By this way, the helical transformation of the unstructured peptide chains in association with the J-aggregation of PQS may explain the same induced CD pattern. Such a concerted mechanism, however, is unlikely due to the missing CD spectroscopic evidence of helical folding. Instead of development of the double dichroic minima characteristic of the α-helical conformation (Figure 3), only a slight red shift and intensity reduction of the main negative peak could be noticed (Figure 6). The negative CE evolved around 230 nm was also displayed in PBS (Figure 4), where the *α*-helical form of LL-37 is dominant. Therefore, it is not of a peptide origin but stems from an optically active electronic transition of PQS. The C-terminal of LL-37 is rich in disorder-promoting residues including Pro33, Glu36, and Ser37, suggesting the potential persistence of local disorder even under helix-promoting environmental settings. This prediction is in line with NMR studies made on LL-37 adsorbed on the surface of dodecylphosphocholine micelles (Porcelli et al., 2008). While the major portion of the peptide chain adopts helical conformation, the C-terminal residues from 30 through 37 are unstructured. The invariant conformational features of this segment may render it a suitable template to initiate a similar chiral supramolecular organization of PQS molecules both in PBS and in a salt-free Tris-HCl buffer.

Notwithstanding the above, some spectral differences in the CD curves recorded in PBS and Tris-HCl buffer should be noted. At the same PQS concentrations, three and two times more intense CEs were induced in PBS (*cf*. Figure 4 and Figure 5). This might be due to the more uniform conformer population of LL-37 present in PBS where the major fraction of the peptide chains is helical, having a short, unordered C-terminal tail. In this case, the chiral inductive effect of the C-terminal end prevails besides the neutral or supportive contribution of the *α*-helical segments. In contrast, the much more heterogeneous, disordered conformational ensembles of LL-37 characteristic of an anion-depleted medium impair the measure and purity of the supramolecular chiral ordering of J-aggregated PQS units.

To estimate the apparent dissociation constant of PQS–LL-37 interactions, absolute ellipticity values of the CD minimum at 230 nm were plotted against the increasing PQS concentrations achieved during the titrations (Supplementary Figure S2). A nonlinear regression analysis of the data points yielded 290 μM and 1.43 for the *K*_d_ and the Hill coefficient, respectively. The greater than 1 value of the Hill coefficient suggests positive binding cooperativity between the PQS molecules.

### Dynamic Light Scattering

To monitor the PQS-provoked aggregation, dynamic light scattering measurements were performed in PBS solution. No particle formation was detected for the native peptide sample at 37 μM. Titration of LL-37 with PQS induced significant changes in the mean hydrodynamic diameter, resulting in large-sized aggregates (Supplementary Figure S3). Similarly, the shift in the correlation function toward higher decay times refers to the formation of larger assemblies in the micrometer range (Supplementary Figure S4).

## Conclusion

QS is a microbial communication process involving the production, release, and perception of hormone-like small molecules. Since inter- and intraspecies QS signaling systems are central regulators of virulence factors, they are promising targets to develop new therapeutic modalities for both the prevention and treatment of infectious diseases. Among the various potential strategies aiming to interfere with QS, generally termed as quorum quenching, one is the hindering of QS molecules to reach their signal receptors. In analogy with the recently recognized siderophore capturing property of LL-37 (Zsila and Beke-Somfai 2020), our current work demonstrates the entrapment of PQS molecules in co-assemblies formed with this host defense peptide. The characteristic absorption spectroscopic features as well as the strong induced CD bands refer to the formation of chiral supramolecular architectures composed of J-aggregated and monomeric PQS species. Considering the uniform induced CD motifs observed in either the folded or unfolded state of LL-37, the C-terminus enriched in disorder-promoting residues is proposed as the primary PQS binding region. These results give further insight into the pleiotropic, multifaceted host defense mechanisms of the innate immune system and unveil a possible quorum quenching role of LL-37 and potentially other HDPs in attenuating microbial pathogenicity, particularly with respect to the biofilm formation and virulence factors of multidrug-resistant bacterial strains. In this respect, it is to be tested whether other molecules of QS systems could also be prone to interactions with HDPs, an area currently under investigation in our laboratory.

## Data Availability

The original contributions presented in the study are included in the article/Supplementary Material; further inquiries can be directed to the corresponding author/s.

## Abbreviations

CD, circular dichroism; CE, Cotton effect; QS, quorum sensing; PQS, *Pseudomonas* quinolone signal.
