# Supplementary material for: Quorum Sensing Pseudomonas Quinolone Signal Forms Chiral Supramolecular Assemblies With the Host Defense Peptide LL-37
Source: Front Mol Biosci. 2021 Oct 11;8:742023. doi: 10.3389/fmolb.2021.742023 (PMC8542694; doi:10.3389/fmolb.2021.742023)
Supplement: Supplementary file 1 [file DataSheet1.PDF]

Quorum sensing *Pseudomonas* quinolone signal forms chiral supramolecular assemblies with the host defense peptide LL-37

Ferenc Zsila, Maria Ricci, Imola Cs. Szigyártó, Priyanka Singh, Tamás Beke-Somfai

Institute of Materials and Environmental Chemistry,

Research Centre for Natural Sciences, P.O. Box 286, H-1519, Budapest, Hungary

Electronic Supplementary Information

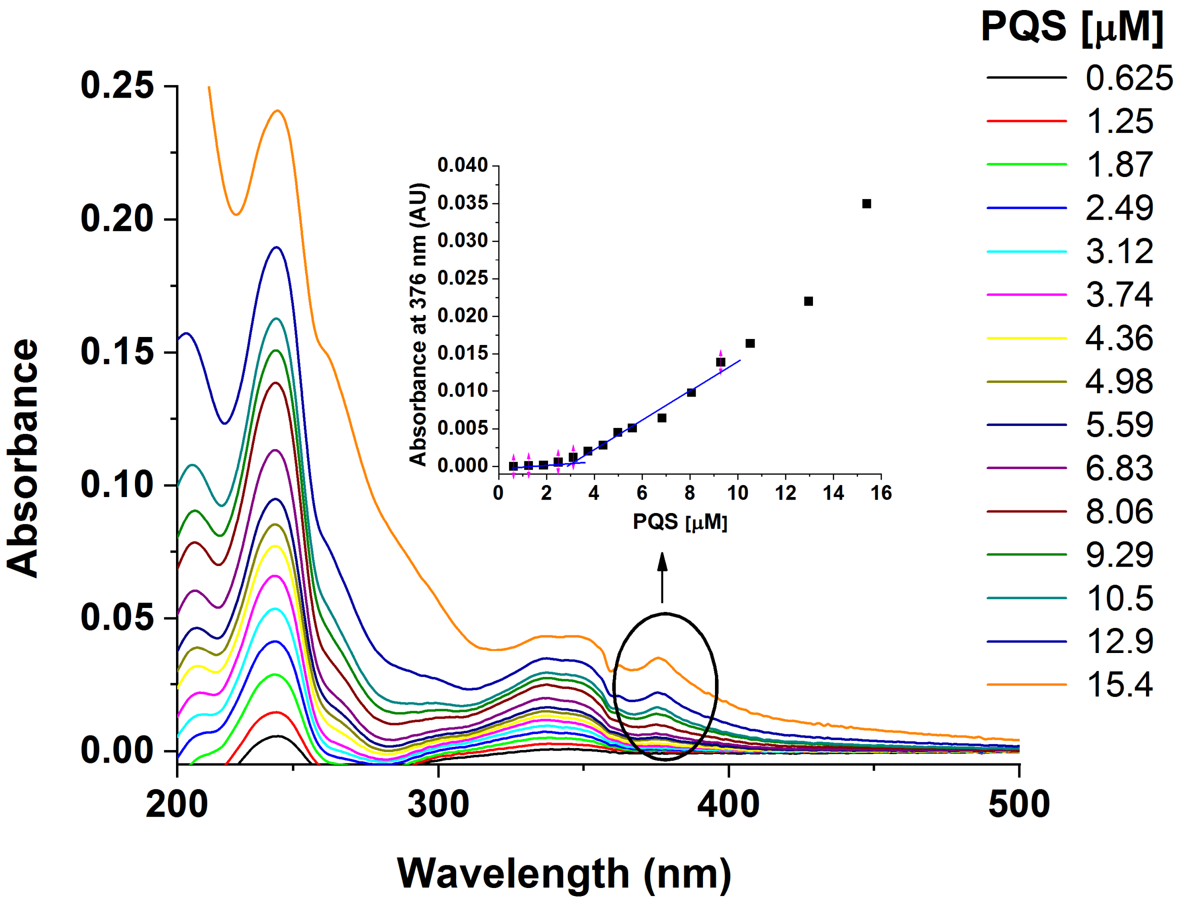

Figure S1.

UV/VIS titration spectra of PQS at increasing concentrations in PBS solution. Inset: absorbance values of the J-band plotted against the ligand concentration. Solid lines are the result of linear curve fitting analysis to determine the critical self-association concentration of PQS (~3 μM).

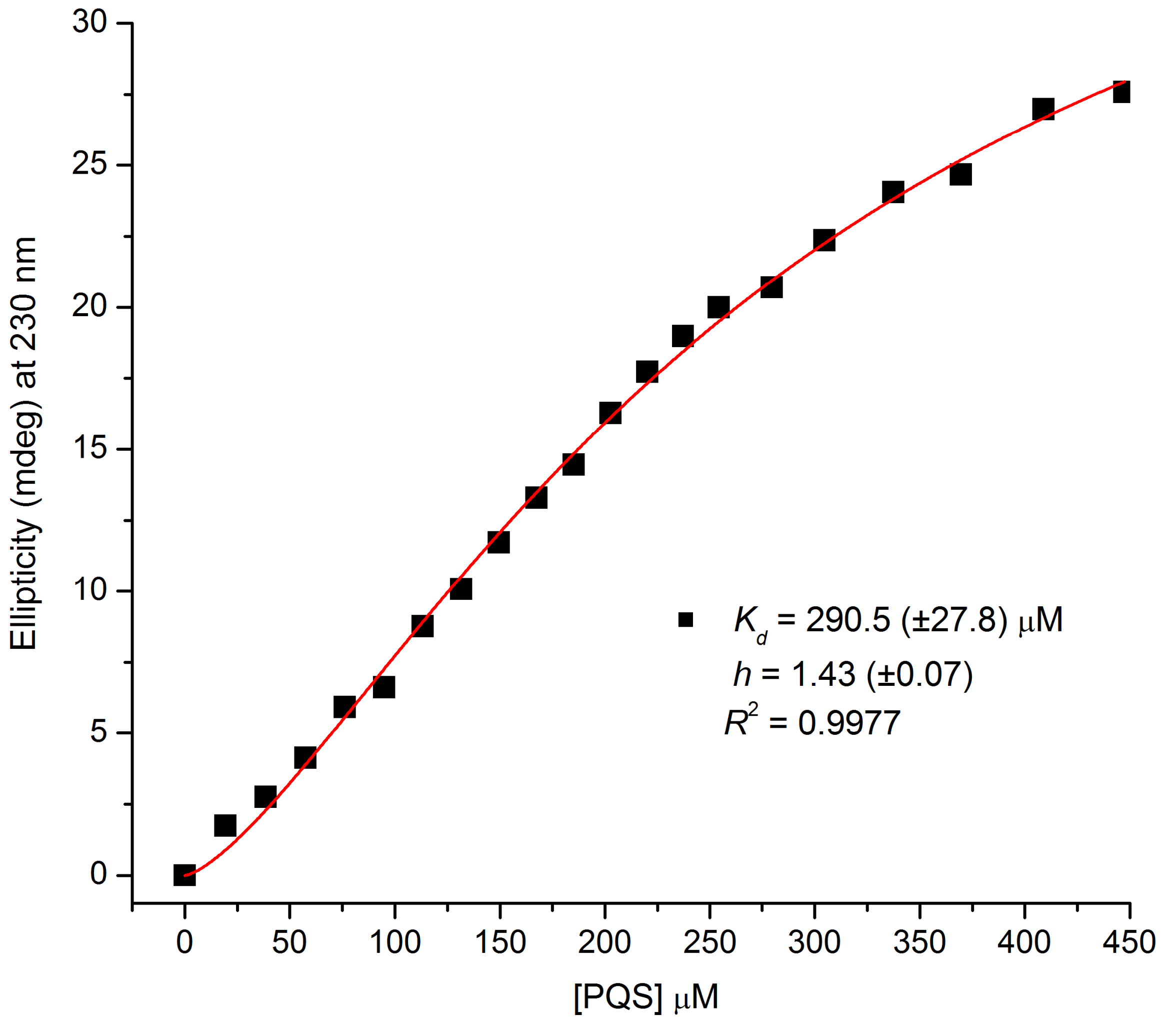

Figure S2.

Non-linear regression analyis of the CD titration data of LL-37 obtained with PQS. Solid line is the result of non-linear curve fitting analysis performed by using the "One site - specific binding with Hill slope" equation built in the Graph Pad Prism software (ver. 6.01, San Diego, California, USA). The estimated K_d value and Hill coefficient (h) are shown.

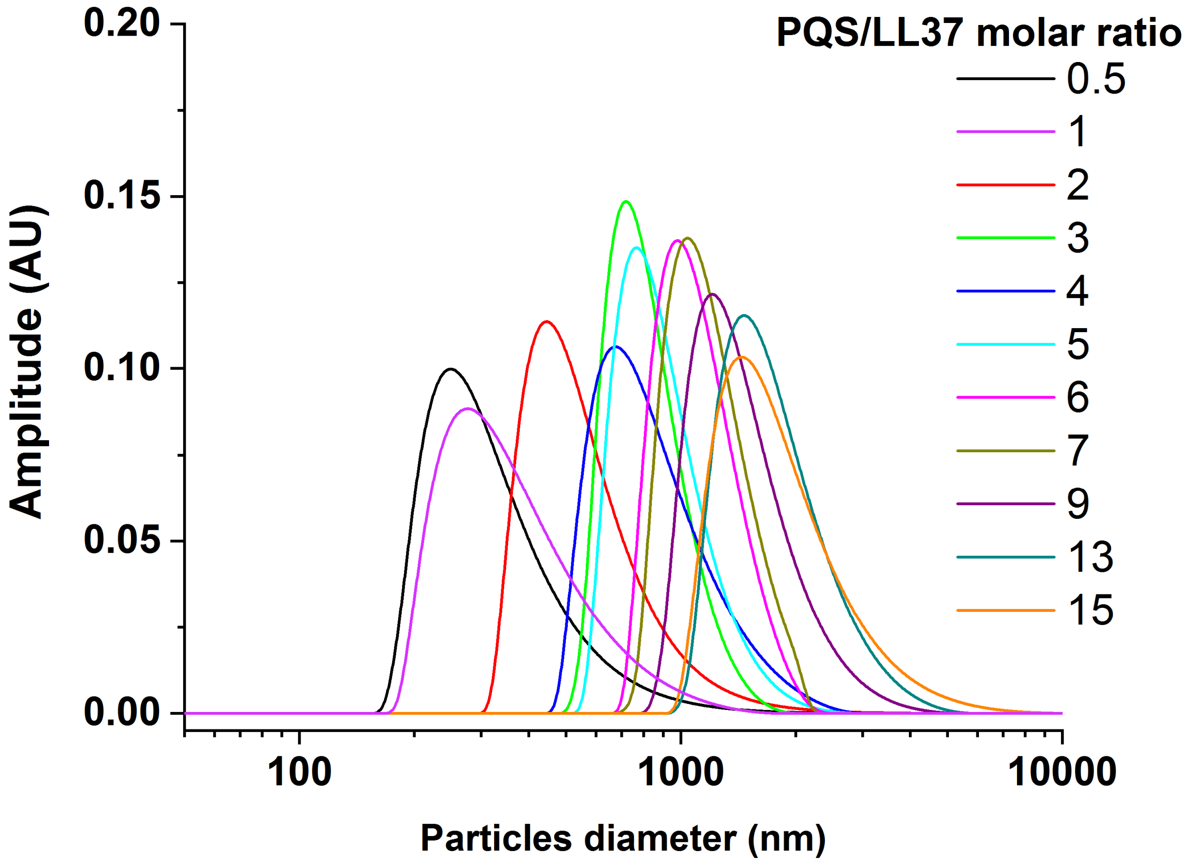

Figure S3.

Size distribution of peptide-PQS aggregates in PBS solution upon stepwise addition of the ligand. LL-37 concentration is 37 μM.

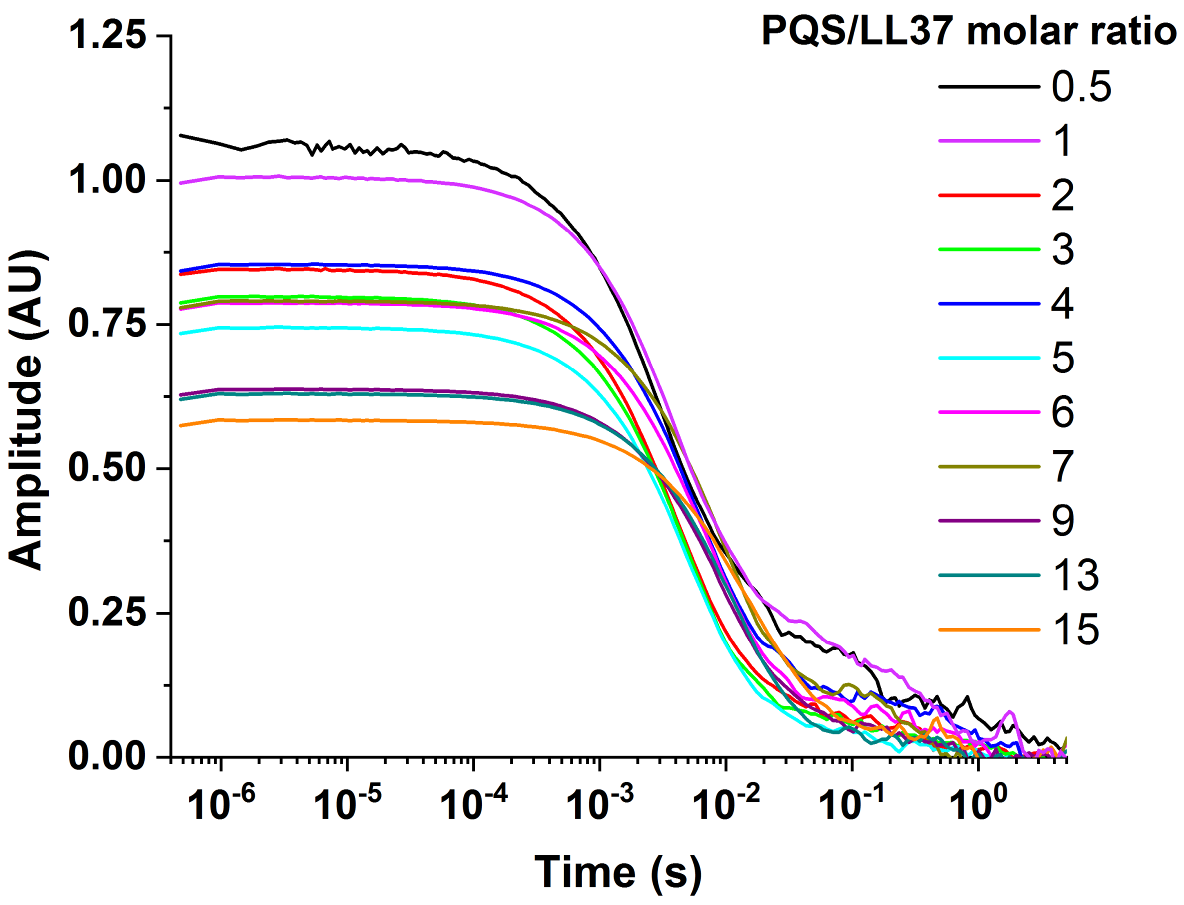

Figure S4.

Autocorrelation function of LL-37 at different PQS concentrations.
